# Evaluation of the effect of items’ format and type on psychometric properties of sixth year pharmacy students clinical clerkship assessment items

**DOI:** 10.1186/s12909-020-02107-3

**Published:** 2020-06-12

**Authors:** Hatim S. AlKhatib, Gayle Brazeau, Amal Akour, Suha A. Almuhaissen

**Affiliations:** 1 Department of Pharmaceutics and Pharmaceutical Technology, School of Pharmacy, The University of Jordan, Queen Rania Street, Amman, 11942 Jordan; 2 Department of Pharmaceutical Sciences, School of Pharmacy, Marshall University, Huntington, West Virginia USA; 3 Department of Biopharmaceutics and Clinical Pharmacy, School of Pharmacy, The University of Jordan, Amman, Jordan

**Keywords:** Assessment items, Clinical clerkships, Difficulty index, Discriminating index, Point Biserial

## Abstract

**Background:**

Examinations are the traditional assessment tools. In addition to measurement of learning, exams are used to guide the improvement of academic programs. The current study attempted to evaluate the quality of assessment items of sixth year clinical clerkships examinations as a function of assessment items format and type/structure and to assess the effect of the number of response choices on the characteristics of MCQs as assessment items**.**

**Methods:**

A total of 173 assessment items used in the examinations of sixth year clinical clerkships of a PharmD program were included. Items were classified as case based or noncase based and as MCQs or open-ended. The psychometric characteristics of the items were studied as a function of the Bloom’s levels addressed, item format, and number of choices in MCQs.

**Results:**

Items addressing analysis skills were more difficult. No differences were found between case based and noncase based items in terms of their difficulty, with a slightly better discrimination in the latter**.** Open-ended items were easier, yet more discriminative. MCQs with higher number of options were easier. Open-ended questions were significantly more discriminative in comparison to MCQs as case based items while they were more discriminative as noncase based items.

**Conclusion:**

Item formats, structure, and number of options in MCQs significantly affected the psychometric properties of the studied items. Noncase based items and open-ended items were easier and more discriminative than case based items and MCQs, respectively. Examination items should be prepared considering the above characteristics to improve their psychometric properties and maximize their usefulness.

## Background

Examinations are the traditional evaluation method of students’ performance used by instructors throughout educational history [1]. Good quality examinations are essential for generating reliable data to measure student learning, guide program improvements and provide stakeholders with relevant information [2]. This places a particularly significant responsibility on educators attempting to develop appropriate examinations’ items [3].

The Accreditation Council for Pharmacy Education (ACPE) standards for the Doctor of Pharmacy (PharmD) programs recommends the implementation of an extensive assessment plan to prepare graduates for practice [4]. A plan should include the use of standardized, systematic, reliable, and valid assessment. It also requires both knowledge and performance evaluation and measurement of the achieved professional competencies.

The quality of assessments is usually expressed in terms of their validity and reliability [5]. However, the quality of tests may be also inferred, at least partially, from the analysis of test items [6].

Consequently, it is essential to analyze and evaluate the assessment items after application. Such analysis and evaluation are needed to improve the items and specify the assessment characteristics; whether the item is performance-oriented, the thinking order the item evaluates, and the item real-life context [7].

Assessment can be performed using different items formats and types; however, assessment items should be developed to address the expected position of a PharmD graduate in healthcare team [8]. The assessment items could be classified according to their format as either case based or noncase based. An item belonging to either format category could be further classified depending on its type/structure as Multiple Choice Questions (MCQs) or an open ended/constructed response question [9, 10].

Case based evaluation items format have the distinct advantage over noncase based format as it can simulate realistic decision-making scenarios and allow student to attempt to solve problems and find alternative plans using individualized detailed information [1, 3, 11]. MCQs are a popular assessment item type where the examinee has to choose the correct answer to the “stem” question from multiple possible answers. Properly constructed MCQs allow the examiner to serve and cover a variety of learning objectives [3, 9, 12]. On the other hand, an evaluation that depends on answering open ended/ constructed response items, allows the exploration of various alternatives rather than concentrating on one correct answer, and encasement of higher thinking orders [3].

National Pharmacy Licensure Examination (NAPLEX) is a health profession examination that measures a candidate’s knowledge of the practice of pharmacy [1]. NAPLEX utilizes assessment items of different formats and types, including constructed response/open ended, and MCQs (A-type, K-type, true-false items, etc.) [13] with the case based type as the most prevalent item format [3].

While the quality of assessment items of different formats and types (case based learning, MCQs, and open-ended items) has been addressed by many authors [3, 10, 11, 14–18], there are only two studies [3, 19] that attempted to compare the quality of case based and noncase based assessment items.

This study evaluated the quality of test items of sixth year clinical clerkships examinations. The examinations were developed, based on revised Bloom’s levels, by a panel of teaching and assessment experts with not less than 5 years of experience in each specific clinical clerkship. The quality of the assessment items as a function of item format (case based versus noncase based) and assessment item type/structure (MCQs and open-ended/essay items) was investigated as well as the effect of the number of response choices on the characteristics of MCQs items.

## Methods

### Data collection

Assessment items used in paper-based examinations of six clinical clerkship rotations (Cardiology, Critical Care, Respiratory, Endocrinology, Oncology, and Nephrology) of the sixth/senior year PharmD program offered by the School of Pharmacy at the University of Jordan, (SP-UJ) were collected. All examinations were final examinations that were 60 to 75 min in length. The examinations were offered in the first and second semesters of the academic year 2015–2016. Each examination was constructed and reviewed by a panel composed of an academic adviser/rotation coordinator, an academic staff member, and at least two preceptors.

A total of 173 assessment items were included in this study. No item was excluded. The student names and University ID numbers were covered to maintain confidentiality.

Assessment items were mapped to Bloom’s educational learning objectives levels. Each item covered one of the Bloom’s levels (Remembering and understanding skills, analysis skills, Application skills and evaluation and creation skills) [20].

Each item was reviewed and categorized as either case based or noncase based by the authors. Case based items were those that were asked in a scenario-based format (i.e. patient profiles with accompanying test questions) so that in order to properly analyze and answer, a student must refer to the information provided in the patient profile [3]. While noncase based “stand-alone questions” had answers that could be drawn solely from the information provided in the question stem [3]. Items were further classified according to their type as MCQs or open-ended (essay) item. MCQs were further classified according to the number of answer options.

The examinations were characterized in terms of their reliability using Cronbach’s Alpha [21] and individual items were characterized in terms of their level of difficulty, discrimination index, point biserial, and number of options (for MCQs) [9, 11, 12, 14, 16, 18, 22, 23]. Individual items or sub-item grades were entered to SPSS (IBM, Armonk, NY) and psychometric parameters were estimated. Item performance/psychometric characteristics calculated included Difficulty Index (difficulty), Discriminating Index (discrimination), and point biserial. The values of these psychometric parameters were used to classify the item quality (Table 1) [6, 12, 15, 16, 23]. Difficulty was calculated as the percentage of the correct responses (MCQs) or the average grade of the specific item relative to the total mark assigned for the item (open-ended). The desired value for difficulty ranges from 20 to 30% at the lower limit, to 75–80% at the higher limit [12]. Discrimination represents the difference between the average grade of the students in the upper quartile (students with highest totals) relative to the item total grade and the average item grade of the students in the lower quartile (students with lowest totals) relative to the item total grade divided by the number of students in a quartile. Point biserial is also a measure of the item discriminative power, this indicator is a comparison of performance on an item relative to whole test performance [6, 12, 22, 24, 25]. Point biserial was estimated using SPSS reliability output [6, 12, 25]. Discrimination and point biserial values can range from − 1 to 1. High values of discrimination and point biserial indicate that an item was correctly answered by high-performing students, and/or incorrectly answered by low-performing students. On the other side, low or negative indices reveal that an item was incorrectly answered by high-performing students, and/or correctly answered by low-performing students; suggesting a poor or flawed item, or poor ability to differentiate between students.

**Table 1:** Descriptive Statistics for Evaluated Assessment Items (*N* = 173)

Variable	No. (%)
Format	
Case based item	134 (77.5)
Non-case based item	39 (22.5)
Type/Structure	
Open-ended/essay item	98 (56.6)
Multiple choice item	75 (43.4)
Number of Choices*	
2 (True/False)	11 (14.7)
3	3 (4)
4	33 (44)
5	28 (37.3)
Bloom’s level	
Remembering and Understanding Skills	60 (34.7)
Analysis skills	57 (32.9)
Application Skills	28 (16.2)
Evaluation and Creation Skills	28 (16.2)
Difficulty Index (Difficulty) Levels^1^	
Difficult (Difficulty< 20%)	6 (3.5)
Acceptable/Good (20% ≤ Difficulty< 50%)	29 (16.7)
Excellent (50 ≤ Difficulty< 80%)	93 (53.8)
Easy/Poor (Difficulty≥80%)	45 (26)
Discriminating Index (Discrimination) Levels^1^	
Poor/Flawed (Discrimination< 0)	5 (2.9)
Poor (0 ≤ Discrimination<.2)	36 (20.8)
Acceptable (.2 ≤ Discrimination<.3)	40 (23.1)
Good (.3 ≤ Discrimination<.4)	39 (22.5)
Excellent (Discrimination≥.4)	53 (30.6)
Point-Biserial Levels^2^	
Poor/Flawed (Point biserial < 0)	14 (8.1)
Poor (0 ≤ Point biserial <.15)	28 (16.2)
Recommended (.15 ≤ Point biserial <.25)	28 (16.2)
Good (Point biserial ≥.25)	103 (59.5)

* Percent relative to MCQs count

### Statistical analysis

The differences in means of item performance characteristics as a function of the items format and type and their interactions were built on two-way Analysis of Variance (ANOVA). Additionally, item performance characteristics as a function of the items format and type were assessed using linear regression. A one – way Analysis of Variance (ANOVA) was used to study the effect of the four Bloom’s levels on the difficulty and discrimination indices. This was followed up by the post-hoc Bonferroni correction test and analysis of variance (ANOVA) on the dependent variables for pairwise comparisons with *p-value < .05* indicating statistical significance. All data analysis was performed using SPSS® 23.0 (IBM, Armonk, NY).

## Results

A total of a hundred and seventy-three items, each answered by 72–83 students were evaluated. These items were collected from 6 different final examinations of clinical clerkships during the senior year of PharmD program offered by the SP-UJ. The reliability of each of the studied examinations as measured using by Cronbach’s Alpha which ranged between .62–.80. The reliability of the studied items differed according to item type; MCQs had an average Cronbach’s Alpha of .61, while for open ended items the average Cronbach’s Alpha was .27. A significant positive Pearson’s correlation was observed between the reliability of each examination measured as the difference between Cronbach’s Alpha value of the examination and Cronbach’s Alpha value if a specific item was deleted and item psychometric parameters; difficulty (*r* = .16, *p < .05*), discrimination (*r* = .63, *p < .001*), and point biserial (*r* = .85, *p < .001*).

Table 1 shows the characteristics of the studied assessment items. Over three-quarters of the items studied (77.5%) were case based. More than half of the items were of open-ended structure that measured students’ remembering and understanding skills. The psychometric parameters of the sample items analysis showed that 54% of the questions had excellent difficulty index (difficulty range 20–50%) [10, 12, 15], almost one third had excellent DI, and around 60% of the questions were on the higher end of point biserial range, while 8.1% of the items had point biserial values below the recommended levels.

Table 2 shows the mean and standard deviation values of difficulty, discrimination, and point biserial for the questions addressing the four Bloom’s levels: remembering and understanding skills, analysis skills, application skills and evaluation and creation skills) as well as one-way ANOVA and follow-up tests results.

**Table 2:** Item Performance Characteristics Based on Item measured ILO Level

Performance Characteristics	Remembering and Understanding Skills Mean (SD)	Analysis Skills Mean (SD)	Application Skills Mean (SD)	Evaluation and Creation Skills Mean (SD)
Level of Difficulty^a^	68.2 (13.3)^b^	56.4 (21.8)	63.2 (20.4)	63.2 (22.4)
Discriminating Index^c^	.41 (.19)^d^	.40 (.18)^d^	.22 (.15)	.25 (.10)
Point biserial^e^	.39 (.16)^d^	.38 (.16)^d^	.21 (.14)	.23 (.14)

*ILO* Intended Learning Outcome

*N* 173

^a^ Model is significant, F (3,169) = 3.7, *p* = 0.012

^b^ significant difference between pairwise comparisons over Analysis skills

^c^ Model is significant, F (3,169) = 12.7, *p < 0.001*

^d^ significant difference between pairwise comparisons over application skills and evaluation and creation skills

^c^ Model is significant, F (3,169) = 14.4, *p < 0.001*

Upon applying one-way ANOVA analysis, significant models (*p < .05*) were found in item characteristics (difficulty, discrimination, and point biserial) as a function of the measured Bloom’s level.

As follow up tests to one-way ANOVA, we performed analysis of variance on the dependent variables (difficulty, discrimination, and point biserial), using Bonferroni method.

The post-hoc analysis to ANOVA for difficulty, discrimination, and point biserial included performing pairwise comparisons. Difficulty of remembering and understanding level items was only significantly higher (*p = .006*) when compared to analysis skills. However, discrimination and point biserial of remembering skills and analysis skills were significantly higher (*p < .001*) than the same metric for application and evaluation and creation levels.

Table 3 represents the analysis of item performance characteristics as a function of different item properties. Case based items were not different in their performance characteristics in comparison to noncase based items. Open-ended type items demonstrated significantly higher discrimination (*p = .006*), and point biserial (*p < .001*) relative to MCQs. On the other hand, 4-option MCQs showed significantly lower difficulty (*p = .013*), but they were not different with respect to discrimination and point biserial; suggesting similar discrimination power.

**Table 3:** Item Performance Characteristics Based on Different Item Properties and Layers (*N* = 173)

	Level of Difficulty Mean (SD)	Discriminating Index Mean (SD)	Point-biserial Mean (SD)
Item Format			
Case Based	63.7 (19.7)	.34 (.18)	0.34 (.17)
Noncase Based	59.2 (19)^NS^	.38 (.22)^NS^	0.30 (.16)^NS^
Item Type/Structure			
Open-ended/Essay	63.4 (18)	.39 (.18)	.39 (.15)
Multiple Choice	61.7 (21.5)^NS^	.31 (.19)*	.26 (.16)*
Number of Choices/options			
4-option Items	51.3 (19.1)	.30 (.18)	.24 (.14)
5-option Items	67.2 (21.5)*	.31 (.19)^NS^	.29 (.17)^NS^
Item Format: Item Type/Structure			
Case Based: Open-ended	64.5 (18.3)	.37 (.17)	.38 (.16)
Case Based: MCQs	62.2 (22.2)^NS^	.29 (.17)*	.26 (.15)*
Noncase Based: Open-ended	54.2 (11.7)	.54 (.17)	.45 (.07)
Noncase Based: MCQs	61 (20.8)^NS^	.33 (.21)*	.25 (.18)*
Item Format: Number of Choices/options			
Case Based: 4-option	50.3 (20.4)	.3 (.17)	.25 (.13)
Case Based: 5-option	73.7 (18.8)*	.27 (.16)^NS^	.29 (.17)^NS^
Noncase Based: 4-option	53.5 (16.6)	.33 (.22)	.21 (.17)
Noncase Based: 5-option	59 (22.6)*	.36 (.23) ^NS^	.28 (.19)^NS^
Item Type/Structure: Item Format			
Open-ended: Case Based	64.5 (18.3)	.37 (.17)	.38 (.16)
Open-ended: Noncase Based	54.2 (11.8)^NS^	.54 (.17)*	.45 (.07)*
MCQ: Case Based	62.2 (22.2)	.29 (.1)	.20 (.1)
MCQ: Noncase Based	61 (20.8)^NS^	.33 (.1)*	.26 (.1)*
Number of Choices/options: Item Format			
4-option: Case Based	50.3 (20)	.3 (.17)	.25 (.13)
4-option: Noncase Based	53.6 (17)*	.33 (.22)^NS^	.21 (.17)^NS^
5-option: Case Based	73.7 (18.8)	.27 (.16)	.29 (.17)
5-option: Noncase Based	60 (22.5)*	.36 (.23)^NS^	.28 (.16)^NS^

*NS* Not Significant

* Significant at measurement level

When items were compared based on item type/structure; case based item that are open ended type showed significantly higher discrimination (*p = .001*), and point biserial (*p < .001*) when compared to case based item that are of the MCQs type. Also, noncase based items, which are open ended type had significantly higher discrimination (*p = .001*) and point biserial (*p < .001*). The number of choices that are case based item possessed a significant impact on the difficulty (*p = .003*), and no effect on discrimination and point biserial. The same effect has been shown by the number of choices on noncase based questions. Open ended items when formatted as case based had no impact on difficulty, but significantly lower discrimination (*p = .001*) and point biserial (*p < .001*). While MCQs comparison based on item format showed higher discrimination measured by discrimination (*p = .001*) and point biserial (*p = .001*) for noncase based items.

MCQ items with four answer options showed significant differences when categorized as case based and noncase based items, this showed as higher difficulty (*p = .003*) and no effect on discrimination. This was not the case dealing with 5 options items as these items demonstrated higher difficulty (*p = .003*) of case based items but no effect of item format on discrimination and point biserial.

Table 4 show linear regression analysis of items performance characteristics in relation to items properties. Regression analysis showed that the Difficulty of an item is not affected by the type of item being open ended or MCQ item, the item format as case or noncase based the linear interactions between them. The same results observed for the item format as case or noncase based, number of choices in MCQ items, and the linear interactions between them. On the other hand, significant model for discriminating index that was affected by both the item type and the item format while the significant point biserial model is only significantly affected by the type of item being open ended or MCQ item, but not the interaction between these factors.

**Table 4:** Linear Regression Analysis of Item Performance as a function of Item Characteristics

Predictor*	Difficulty Index		Discrimination Index		Point biserial	
	Coefficient	*p-value*	Coefficient	*p-value*	Coefficient	*p-value*
Model 1^$#^	F(2,170) = .78	.46	F(2,170) = 6.84	.001	F(2,170) = 15.192	<.001
Case/Noncase based items	−.04	.26	.08	.019	.01	.67
Open-ended (essay)/MCQs items	−.01	.89	−.10	.001	−.14	<.001
Model 2^@#^	F(2,72) = .76	.47	F(2,72) = .40	.67	F(2,72) = .18	.83
Case/Noncase based items	−.02	.77	.04	.39	−.01	.79
Number of Choices	−.03	.23	.01	.78	.01	.60

*because the number of choices variable can’t be included in the same model as the item type variable, two separate models (one for item format and item type and then one for item format and number of choices among the MCQ questions) were analyzed

^$^*N* = 173

^@^*N* = 75

^#^The interaction of the two factors in the model was tested and no significant effect was detected with also no effect on the significance on other factors

## Discussion

The present study addressed the quality of assessment items in sixth year PharmD clinical clerkships examinations. The study provided three interesting and valuable outcomes that can be of benefit to academic staff and preceptors. (1) The reliability of an examination correlated significantly with items psychometric parameters, (2) the Bloom’s levels associated with an item significantly affected its psychometric properties, and the (3) structure of an item and the number of options possessed by an item significantly affected the psychometric parameters of the item.

The predominant item format in the current study was case based, in which the basic level competencies; remembering and understanding skills, constituted the majority; around two-thirds, of the measured skills. These competencies are the foundation for the higher competencies levels (e.g. analysis, application and evaluation and creation skills). The use of case based items in the assessment of students in a health care professional program, such as a Pharm D program, is necessary. A case based item acts to introduce students to clinical scenarios that simulate patient situation, and enables them to practice decision making during realistic challenges.

Building case based items is a time consuming task and requires a knowledgeable and practice expert examiner [3, 7]. The psychometric parameters of the studied assessment items in our study showed their high quality with less than 8% classified as poor or flawed items [16, 23]. The benefits implied by the use of case based items and items psychometrics parameters, in addition to the high values of Cronbach’s Alpha of each examination evidenced the high reliability of exams under study [21].

Evaluation of the effect of competency levels on the difficulty of an item showed that items addressing analysis skills are more difficult; on the other end of the scale are knowledge and understanding skills which were much easier. These findings are in agreement with the findings of Kim and colleagues (2012), where they found that analysis and synthesis items are more difficult [24].

The evaluation on discrimination measures (discrimination and point biserial) of assessment items addressing remembering and understanding skills and analysis skills are more efficient in differentiating between students in upper and lower grade quartiles.

Analysis on difficulty, discrimination, and point biserial of item formats demonstrated no differences between case based and noncase based items in terms of difficulty, discrimination and point biserial. These results are similar to that of Phipps and Brackbills (2009) findings [3], demonstrating comparable capability of these two item formats.

The type of an item has significant effect on its psychometric characteristics. Open ended type was easier, yet more discriminative; this tallies well with Thawabieh (2016) findings [19]. It is understood that the nature of open ended items allows for the incorporation of more details when answered by students, while utilizing higher thinking orders allows for better discrimination between high- and low-performance students. On the other hand, the options in MCQs may provide a hint to students on the item-writer intention [24].

The number of options an item possessed showed significant impact on difficulty and none on discrimination levels measured as discriminating index and point biserial. The higher the number of options the easier the item is and, slightly but not significantly, more discriminative. This is in partial agreement with Phipps and Brackbills (2009) findings where they found that 5-options are more difficult and more discriminative. Despite that, they concluded that due to the very small differences between these two groups, it is explainable/justifiable to use a mix of 4 and 5 responses MCQs in exams [3].

Analyzing case based items and noncase based items separately revealed different behaviors. Case based items that are open ended are significantly easier and more discriminative than MCQs, while the same type of noncase based items is more difficult and more discriminative. This can be attributed to the fact that case based items provide scenarios that may simplify the item and guide the examinee but still need to be seen in context.

The number of answer options (4 or 5), had no effect on discrimination metrics of either case based or noncase based assessment items, and it only affected the difficulty of case based items, as 4-option questions were more difficult. The idea of writing more plausible and effective options other than the key answer when an item is based on a case that’s full of details is clearly more challenging and difficult.

Open ended items that are noncase based are slightly difficult and more discriminative in comparison with open ended that are case based. In addition, MCQs that are noncase based have larger discrimination and point biserial; showing that noncase based items are more discriminative. Again, case based items were shown to have similar, if not inferior, behavior to noncase items, limiting their benefit to their ability to address intended learning and course aims, but expressing no unique performance assessment characteristics.

One more result of the current study was the comparable effect of the item format on the characteristics of 4- and 5-option MCQs. Noncase based, 4-option MCQs items were significantly easier than case based 4-option MCQs with similar discriminative power. However, case based and noncase based 5-option MCQs items had no differences in discrimination and differed slightly in difficulty as case based items are easier.

The previous results showed differences between the two MCQs groups yet cannot be conclusive, as it once again a very challenging time-consuming task not only to construct a case item but also to construct strong, reliable, and efficient choices during the creation of MCQs regardless the item format; being based on case or not.

In a study conducted by Sheaffer and Addo (2013), where they measure both second year Pharm D students’ performance and confidence in answering selected-response and constructed-response items, it was concluded that students performed better and felt more confident in answering selected-response items. Moreover, the incorporation of constructed-response teaching and testing method in pharmacy learning and education was recommended [13].

It is understandable in a study like ours that items classification based on the Bloom’s levels might be subjective [8]; we have attempted to minimize that by making use of the experience of clinical preceptors in direct contact with “real life” cases and academic staff/educators as peer reviewers of the studied items. Another issue of importance to consider in the present study is the fact that we had unequal number of items per rotation could have an effect on the analysis.

The current study based its analysis on the Classical Test Theory; it would be interesting and useful to attempt to utilize alternative approaches to evaluate the properties of items such as the Item Response Theory which is based on the study of test and item scores based on assumptions concerning the mathematical relationships between abilities and item responses [26]. Another potentially useful analytical approach involves testing Bloom’s levels and item properties in the same model which would be attempted in future studies. It would also be of great value to include.

One last important limitation of the current study is the use of Cronbach’s alpha as the only measure of exam internal consistency which could be affected the number of items in each of the tested examinations. An alternative approach would be the supplementation of Cronbach’s alpha with other indices of internal consistency.

## Conclusion

Reliable and effective assessment of students in health care professional programs where decisions related to patients’ treatment are to be made is crucial. PharmD students should be trained to deal with real medical cases during their study course especially senior year. Psychometrics parameters are efficient in evaluating clerkships examinations items. The study showed that the psychometric properties of items is dependent on the associated Bloom’s levels. Item formats, structure, and number of options in MCQs, as well as the different combinations of these factors affected the psychometric properties of items and the value of Cronbach’s alpha. The necessity to build examination that are able to measure student learning and contribute to programs development is daunting. It is critical to develop training programs for educators on how to construct “good” items and examinations.

## Supplementary information

**Additional file 1.**

## Data Availability

The datasets used and analyzed during the current study are available from the corresponding author on reasonable request.

## Abbreviations

ACPE: Accreditation Council for Pharmacy Education
PharmD: Doctor of Pharmacy
MCQ: Multiple Choice Questions
NAPLEX: National Pharmacy Licensure Examination
SP-UJ: Pharmacy at the University of Jordan
difficulty: Difficulty Index
discrimination: Discriminating Index
MANOVA: One-way analysis of variance
ANOVA: Analyses of variance
