# Supplementary material for: Evaluation of the effect of items’ format and type on psychometric properties of sixth year pharmacy students clinical clerkship assessment items
Source: BMC Med Educ. 2020 Jun 12;20:190. doi: 10.1186/s12909-020-02107-3 (PMC7291500; doi:10.1186/s12909-020-02107-3)
Supplement: Supplementary file 1 — Additional file 1. [file 12909_2020_2107_MOESM1_ESM.pdf]

كلية الصيدلة
SCHOOL OF PHARMACY

الجامعة الأردنية
THE UNIVERSITY OF JORDAN

قسم الصيدلة الحيوية والسريرية
Department of Biopharmaceutics
& Clinical Pharmacy

No.: .... :الرقم

Date: .... :التاريخ

December 05, 2017

Suha A. Al Muhaissen, MSc,
Lecturer, Department of Pharmaceutics and Pharmaceutical Technology
School of Pharmacy, The University of Jordan

Dear Ms. Suha Al Muhaissen,

I would like to inform you that the Scientific Research Committee in the School of Pharmacy at The University of Jordan has reviewed your study plan titled: "Evaluation of the Effect of Items' Format and Type on Psychometric Properties of Clinical Clerkship Assessment Tools".

Your study was accordingly granted an exemption from IRB review based on the fact that "the information is recorded in a manner that individuals cannot be identified (directly or through identifiers linked to the individual)"

Best regards,

Prof. Violet Kasabri,
Chairwoman, Scientific Research Committee,
School of Pharmacy, The University of Jordan

هاتف- ٥٣٥٥٠٠٠ (٦-٩٦٢) فرعي- ٢٣٣٥٥ فاكس- ٥٣٠٠٢٥٠ (٦-٩٦٢) عمان ١١٩٤٢ الأردن
Tel.: (962-6)5355000 Ext.: 23355 Fax: (962-6) 5300250 Amman 11942 Jordan
E-mail: deanphar@ju.edu.jo
